# One-step multiplex real time RT-PCR for the detection of bovine respiratory syncytial virus, bovine herpesvirus 1 and bovine parainfluenza virus 3

**DOI:** 10.1186/1746-6148-8-37

**Published:** 2012-03-28

**Authors:** Leenadevi Thonur, Madeleine Maley, Janice Gilray, Tara Crook, Ellie Laming, Dylan Turnbull, Mintu Nath, Kim Willoughby

**Affiliations:** 1 Moredun Research Institute, International Research Centre, Pentlands Science Park, Bush Loan, Penicuik, Midlothian EH26 0PZ, UK; 2 Biomathematics and Statistics Scotland, James Clerk Maxwell Building, The King's Buildings, Edinburgh EH9 3JZ, UK

## Abstract

**Background:**

Detection of respiratory viruses in veterinary species has traditionally relied on virus detection by isolation or immunofluorescence and/or detection of circulating antibody using ELISA or serum neutralising antibody tests. Multiplex real time PCR is increasingly used to diagnose respiratory viruses in humans and has proved to be superior to traditional methods. Bovine respiratory disease (BRD) is one of the most common causes of morbidity and mortality in housed cattle and virus infections can play a major role. We describe here a one step multiplex reverse transcriptase quantitative polymerase chain reaction (mRT-qPCR) to detect the viruses commonly implicated in BRD.

**Results:**

A mRT-qPCR assay was developed and optimised for the simultaneous detection of bovine respiratory syncytial virus (BRSV), bovine herpes virus type 1 (BoHV-1) and bovine parainfluenza virus type 3 (BPI3 i & ii) nucleic acids in clinical samples from cattle. The assay targets the highly conserved glycoprotein B gene of BoHV-1, nucleocapsid gene of BRSV and nucleoprotein gene of BPI3. This mRT-qPCR assay was assessed for sensitivity, specificity and repeatability using *in vitro *transcribed RNA and recent field isolates. For clinical validation, 541 samples from clinically affected animals were tested and mRT-qPCR result compared to those obtained by conventional testing using virus isolation (VI) and/or indirect fluorescent antibody test (IFAT).

**Conclusions:**

The mRT-qPCR assay was rapid, highly repeatable, specific and had a sensitivity of 97% in detecting 10^2 ^copies of BRSV, BoHV-1 and BPI3 i & ii. This is the first mRT-qPCR developed to detect the three primary viral agents of BRD and the first multiplex designed using locked nucleic acid (LNA), minor groove binding (MGB) and TaqMan probes in one reaction mix. This test was more sensitive than both VI and IFAT and can replace the aforesaid methods for virus detection during outbreaks of BRD.

## Background

Bovine respiratory disease (BRD) is a major disease problem for the cattle industry, causing huge economic losses; research on BRD has been a longstanding global priority. While the aetiology is multifactorial, infectious agents are important in the development of disease. The important infectious causes of BRD include viruses, bacteria and mycoplasma [1,2]. Aside of infectious causes, stress and environmental factors such as weaning, temperature, stocking density, dust, humidity, transportation and inadequate nutrition are also important co-factors in development of disease [3]. In economic terms, BRD leads to decreased production, higher levels of mortality and morbidity, increased veterinary and labour costs and reduced carcass value [4-6].

The primary viral respiratory pathogens are bovine herpesvirus-1 (BoHV-1, also known as bovine infectious rhinotracheitis virus), bovine respiratory syncytial virus (BRSV), and bovine parainfluenza type 3 (BPI3) [1,2]. While usually considered a respiratory pathogen, infection with BoHV-1 can also cause abortion in pregnant cattle [7]. Infection with these viruses can also facilitate invasion of opportunistic secondary pathogens such as *Mannheimia haemolytica, Pasteurella multocida, Haemophilus somni *and a number of mycoplasma species such as *M. bovis *and *M. dispar *[1,2,8]. On farms where bovine viral diarrhoea virus [BVDV] is not well controlled, this can lead to immunosuppression and influence the progression of BRD. Permanent lung damage can result following an episode of BRD, making animals more susceptible to subsequent episodes of respiratory disease compromising growth rates and economic returns for the farmer [4,9,10].

Irrespective of the infectious agent involved, the presenting clinical signs of BRD can appear similar. Moreover, the detection of bacterial pathogen can mask an underlying viral cause; virus isolation may not always be successful and alternative methods of detection like IFAT can lack sensitivity and specificity compared to molecular detection methods. Single target (mono-specific) PCR assays require separate amplification of each target and can be expensive, inefficient and resource intensive where multiple pathogen detection is necessary. In this scenario, multiplex PCR has a significant advantage, as it permits simultaneous detection of several viruses in a single reaction mixture, facilitating cost-effective diagnosis [11]. Real time PCR can provide rapid results for the clinical virologist with a reduced risk of contamination, and can detect, differentiate and provide a quantitative result for many different targets without any single target influencing the detection of the others [12,13].

The aim of this study was to develop a simple, sensitive, specific, rapid and cost effective mRT-qPCR method for the detection of BRSV, BoHV-1 and BPI3 in clinical samples. The assay was compared to the conventional methods of virus isolation and FAT to assess its application in routine diagnosis of the aetiological agents involved in BRD.

## Results

### Integrity of clinical material

A β-actin signal was detected in all clinical samples tested in the mRT-qPCR indicating no evidence of extraction failure or PCR inhibition.

### Specificity of the mRT-qPCR

The performance of the mRT-qPCR on the virus panel demonstrated neither non-specific reactions nor any inter-assay cross amplification. On well characterised archived isolates for each of the viruses (Table 1), only the intended target virus was amplified by the mRT-qPCR.

**Table 1:** Different isolates of BRSV, BoHV-1 and BPI3 tested in this study

BRSV	BoHV-1	BPI3 i	BPI3 ii
A4446/A	6660	N3322	A2084
A4644/3	Naselgen	L3380	J2365
B446/A	Tracherine	L3047/1	A2112
B4332/1	Aberdeen	B2279/3	B788
B4332/2	Oxford		
B4446/A	Stricken		
B4596/1			
B4596/3			
B4596/4			
D4636/1			
D4658/7			
R4017/5			

### Sensitivity of the mRT-qPCR

**Figure 1:**
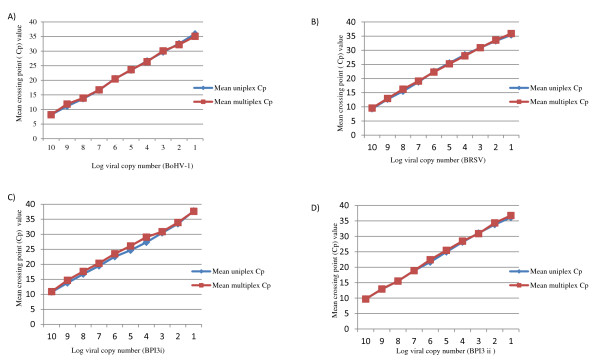
**Comparison of the standard curves of the uniplex and multiplex real time RT-PCR assay for the detection of (A) BoHV-1, (B) BRSV, (C) BPI3 i and (D) BPI3 ii**.

A ten-fold serial dilution of each of the *in vitro *transcribed RNAs was tested in triplicate and the mRT-qPCR assay compared to the same template using the mono-specific assay; similar Cp values were obtained for both formats. The standard curves obtained from the mRT-qPCR and mono-specific assay are shown in Figure 1 A, B, C, D. There was no appreciable difference in the mean Cp values of the mono-specific assay and the mRT-qPCR assay for BoHV-1, BRSV and BPI3. The estimates of Cp values (95% lower, upper confidence intervals) that corresponds to 97% diagnostic test sensitivity conditional on virus copy numbers and test methods for each virus are presented in Table 2. As observed for the standard curve, the Cp values for the mono-specific assay and the mRT-qPCR assays were very similar and hence both assays may be regarded as equally sensitive for diagnostic purpose. These results confirmed that the mRT-qPCR could achieve sensitivity of 97% with Cp values in the range of 31-33 when the samples included 10^3 ^viral genome copy numbers for all targets; a similar sensitivity was shown with Cp values in the range of 34-35 for 10^2 ^viral genome copy numbers. Moreover, the efficiency of the PCR reaction and the detection limits of each target virus were not compromised by multiplexing the reaction (Table 3).

**Table 2:** Estimates (95% lower, upper confidence levels) of mean cut-off Cp values after amplification of a dilution of titrated virus in uniplex and multiplex real time RT-PCR for a test sensitivity of 97%a

		Mean Cut-off Cp value	
**Virus**	**Copy number**	**Uniplex RTPCR**	**Multiplex RTPCR**

BRSV	10^3^	32.9 (32.1, 33.6)	33.0 (32.2, 33.7)
	
	10^2^	35.4 (34.5, 36.1)	35.4 (34.6, 36.1)
	
	10	37.7 (36.8, 38.5)	37.7 (36.9, 38.5)

BoHV-1	10^3^	31.4 (31.0, 31.9)	31.4 (31.0, 31.9)
	
	10^2^	35.2 (34.8, 35.8)	35.2 (34.8, 35.8)
	
	10	37.1 (36.6, 37.6)	37.1 (36.6, 37.6)

BPI3 i	10^3^	31.7 (31.2, 32.4)	32.5 (31.9, 33.1)
	
	10^2^	34.4 (33.9, 35.1)	35.2 (34.6, 35.8)
	
	10	39.4 (39.0, 40.2)	40.2 (39.7, 41.0)

BPI3 ii	10^3^	32.4 (32.2, 32.8)	32.8 (32.6, 33.1)
	
	10^2^	34.7 (34.4, 35.0)	35.1 (34.9, 35.4)
	
	10	38.4 (38.2, 38.8)	38.9 (38.7, 39.2)

^a ^For each virus, a 10-fold dilution series is shown with the mean Cp values. The lowest level of detection is shown, as are the two 10-fold dilutions immediately prior to the lowest level of detection. Each dilution series was tested four times in triplicate. The estimates of the mean cut-off Cp value for a test sensitivity of 97% and corresponding 95% confidence intervals were obtained using a bootstrap sampling procedure (see text for detail).

**Table 3:** Detection limits and efficiencies of the multiplex and uniplex RT-QPCRs

Virus	Detection limit (copy no. per μl)	Reaction efficiency		
	**Multiplex**	**Uniplex**	**Multiplex**	**Uniplex**

BoHV-1	10^1^	10^1^	2.01	1.99

BRSV	10^1^	10^1^	2.14	2.00

BPI3 i	10^2^	10^2^	1.92	1.99

BPI3 ii	10^2^	10^2^	2.09	2.03

### Simultaneous detection of multiple virus targets

The template mixture of ten-fold serial dilutions of the four *in vitro *transcribed virus targets was tested in triplicate by mRT-qPCR assay. All four targets were detected and no evidence of cross reactivity between primers and probes or any reduction in sensitivity was observed.

### Analysis of clinical samples

The performance of the mRT-qPCR assay in clinical material was evaluated on several different sample types including swabs, fresh and fixed tissues and bronchoalveolar lavage samples; further, a small number of tissue culture supernatants (n = 15) derived from clinical samples were included (total samples tested = 541; see Table 4 for sample detail). Results of IFAT and virus isolation were not available for all 541 samples. This is because the testing was targeted depending upon the decisions made by individual private veterinary surgeons and diagnosticians at external veterinary laboratories submitting the material. IFAT was performed on 214 samples for BoHV-1, 222 samples for BRSV and 106 samples for BPI3. Virus isolation was carried out on 80 samples.

**Table 4:** Multiplex real time RT-PCR results for 541 clinical samples

Clinical material	Number of samples tested	BoHV-1 detected	BRSV detected	BPI3 detected
Liver (fetal)	189	2	0	0

BAL	19	4	0	2

Swab	136	27	14	4

Lung	106	5	10	7

Spleen	9	1	0	0

Placenta	32	2	0	0

Fetal fluid	7	0	0	0

Trachea	19	6	1	1

Cell culture	15	1	3	0

Oesophagus	1	0	0	0

Tonsil	1	0	0	0

Brain	1	0	0	0

Wax blocks	6	0	0	0

A list of clinical material positive for BRSV, BPI3 and BoHV-1 is shown.

BoHV-1 nucleic acid was detected as a single agent by the mRT-qPCR in 48/541 samples, BRSV RNA in 28/541 samples and BPI3 i RNA in 14/541 samples; co-infection with BoHV-1 and BPI3 i was observed in 3 samples. While BPI3 ii had been detected previously, it was not identified during this study period.

Of the 48 samples in which BoHV-1 nucleic acids were detected, BoHV-1 was also identified in 23 by IFAT. BoHV-1 was isolated from 12 samples, including 8 samples positive by IFAT; in 4 samples BoHV-1 was isolated but not detected by IFAT. In addition, IFAT detected BoHV-1 in 3 samples where BoHV-1 was not detected by the mRT-qPCR or by virus isolation; this likely indicates poorer specificity of the IFAT for BoHV-1. BoHV-1 was not detected in the remaining samples tested by either IFAT (n = 188) or virus isolation (n = 68).

Of the 28 samples in which BRSV RNA was detected, BRSV was detected in eight samples by FAT; BRSV was not detected by virus isolation in any sample. BRSV is difficult to detect by virus isolation as infectivity is rapidly lost outside the host. BRSV was not detected in any of the remaining samples by FAT (n = 214).

Of the 14 samples in which BPI3i RNA was detected, BPI3 was detected in 3 by virus isolation; but in none of the 106 samples tested by IFAT.

### Repeatability

The estimates of repeatability standard deviation (SD_r_) and relative standard deviation of repeatability (RSD_r_) of uniplex and multiplex assays for different viruses are presented in Table 5. The multiplex assay showed slightly higher estimates of SD_r _and RSD_r _than the uniplex assay for all viruses. The estimates of SD_r _in uniplex assays ranged from 0.39 to 0.58 while for multiplex assays they ranged from 0.70 to 1.30. The estimates of RSD_r _(%) ranged from 1.04 to 1.50 and 1.79 to 3.40 for uniplex and multiplex assays respectively. In general, estimates of SD_r _and RSD_r _for both assays were within the acceptable range of a standard diagnostic test (Table 5).

**Table 5:** Estimates (95% lower, upper confidence levels) of standard deviation (SD_r_) and relative standard deviation (RSD_r_) of repeatability of uniplex and mRT-qPCR assays

Virus	Uniplex		Multiplex	
	**SD_r_**	**RSD_r _(%)**	**SD_r_**	**RSD_r _(%)**

BoHV-1	0.39 (0.31, 0.49)	1.04 (0.82, 1.31)	1.15 (0.94, 1.41)	3.10 (2.53, 3.79)

BRSV	0.46 (0.36, 0.59)	1.26 (0.99, 1.60)	1.30 (1.10, 1.53)	3.40 (2.88, 4.02)

BPI3-i	0.58 (0.29, 1.17)	1.50 (0.75, 3.00)	0.70 (0.59, 0.83)	1.79 (1.50, 2.13)

BPI3-ii	0.57 (0.43, 0.75)	1.36 (1.03, 1.80)	0.86 (0.71, 1.04)	2.07 (1.71, 2.50)

To further investigate the reproducibility and performance of this assay on clinical materials, twenty positive and negative samples each for BRSV and BoHV-1 and fourteen positive and negative samples of BPI3 i by multiplex assay were randomly selected. These samples were re-tested in parallel by both mono-specific and mRT-qPCR assays. Both assay formats identified the appropriate target and the results matched the original mRT-qPCR test result.

## Discussion

The mRT-qPCR is a rapid and efficient method for the detection and differentiation of BRSV, BoHV-1 and BPI3 and is thus an invaluable tool in the aetiological resolution of BRD. Aside of multiple pathogen detection, the assay has several advantages over conventional methods including higher sensitivity and specificity, decreased cost, smaller sample size, rapidity of processing and the possibility of laboratory automation to suit high throughput veterinary diagnostic laboratories. The multiplex format showed complete concordance with the corresponding mono-specific RT-PCRs. The application of mRT-qPCR for the detection of multiple pathogens provides a major contribution to efficiency, logistics, and cost-effectiveness of molecular diagnostics [14-18].

A multiplex real time RT-PCR has been reported previously for BVDV (5'UTR), BoHV-1 (glycoprotein C) and BPI3 (matrix) [18]. While BVDV can be important in the development of BRD, BRSV is a primary pathogen and is thus included in the mRT-qPCR described herein. The use of shorter length MGB and LNA probes is advantageous, typically confering stability, target specificity, greater sensitivity and discrimination for the target gene [19].

As co-infections are a regular feature of BRD in field outbreaks [1,2,10,20], careful optimisation is required to ensure that the molecular diagnostic test employed will not detect one target virus preferentially. Primers and probes for multiplex assays should be assessed both *in silico *and *in vitro *for evidence of cross amplification, competition or inhibition. The mRT-qPCR assay described here can detect viral co-infections both in technical validation experiments and in clinical samples. No cross reactivity between primers and probes was observed, nor was a reduction in sensitivity detected.

The sensitivity of the multiplex and uniplex assay was evaluated by testing 10-fold serial dilutions of in vitro transcribed RNAs for BRSV, BoHV-1 and BPI3 i and ii. The sensitivity, efficiency and detection limits of the individual RT-qPCRs were not affected by multiplexing the reactions. The standard curves and reaction efficiencies were very similar for the mRT-qPCR and mono-specific reactions (Figure 1 A, B, C, D; Table 3). A perfect amplification reaction has an efficiency of 2, but in reality, reactions often have efficiencies of less than2; the acceptable range is considered to be between 1.7 and 2.2 [21]. The efficiencies obtained for both assays in this study were within this range.

For BRSV and BPI3, all samples in which these viruses were detected by other methods were detected by the mRT-qPCR. Additionally, the mRT-qPCR identified BRSV and BPI3i in 20 and 14 additional samples respectively, suggesting a higher sensitivity. For BoHV-1, this was the case for virus isolation and for the majority of FAT positive samples. However, three samples were positive on BoHV-1 FAT but negative on both virus isolation and mRT-qPCR; this suggests a lack of specificity in the FAT; further, the mRT-PCR identified BoHV-1 in 25 additional samples when compared to FAT results, suggesting higher sensitivity. The mRT-qPCR also detected dual infections (BoHV-1 and BPI3 i) in three samples; in virus isolation of these samples, BoHV-1 overgrew BPI3, masking detection. These results demonstrate that the m RT-qPCR is more specific and sensitive in respiratory viral diagnosis when compared to conventional tests, as has been shown in both veterinary and human clinical pathology settings [16,22-25].

False negative results can occur due to RT- PCR inhibition, which was controlled in this sample set by the use of an endogenous internal control (β-actin). While this assay was run separately, it would be possible to consider including this control in the multiplex reaction by, for example, labelling both subgenotypes of BPI3 with the same fluorophore. The selection of target sequences is also critical factor that can contribute to false negatives; it is possible (especially for RNA viruses, due to the higher error rate of RNA polymerases) that mutations in the primer and probe regions may occur which compromise molecular detection at the target site (25). While sequences for molecular assays are selected *in silico *to ensure the target region is highly conserved, unusual or unexpected results from clinical samples should always trigger further investigation by either conventional methods or use of molecular assays with different target sequences or degenerate primers.

A final advantage of the use of molecular based testing in a clinical setting is the ability to include other targets e.g. as adenovirus, BVDV and bacterial pathogens. The limitations of real time PCR based multiplex detection and differentiation rest with the number of reactions which can be optimised in a single tube and the number of fluorophores which can be simultaneously detected. While real time PCR platforms can generally detect no more than 5 fluorophores, labelling strategies can be used to increase the number of targets detected and the development of fluorophore labelled bead based detection systems (e.g. Luminex assays) may extend target detection. Currently, the aetiology of many BRD outbreaks is undiagnosed, in some part due to the range of respiratory pathogens which must be sought and the cost of multiple pathogen detection by mono-specific assays. Adding further pathogen targets to molecular assays should improve aetiologic identification in investigation of BRD.

## Conclusions

In conclusion, this mRT-qPCR assay is a sensitive and specific technique capable of detection of three major viral respiratory pathogens of cattle, and may have additional benefit when more than one agent is involved. Although reagents for multiplex and real time PCR assays are traditionally considered expensive, the ability to perform these assays within a short time frame to detect multiple pathogens reduces hands-on time in the laboratory, is more efficient and can generate valuable information in differential diagnosis. Additional cost benefits on farm will result from more rapid diagnosis and the ability to target treatment, use appropriate vaccines or implement improved management procedures quickly.

## Methods

### Viruses

The viral strains used in this study included isolates of BRSV, BoHV-1, and BPI3 i & ii. A vaccine isolate of BRSV (Rispoval RS, Pfizer Ltd) was used for assay optimisation and eight other BRSV isolates were used for preliminary validation experiments [22,26]. A reference strain of BoHV-1 (6660) was used for the initial optimisation of assay and further five isolates were tested for validation purposes [27]. Preliminary amplification and sequencing studies targeting fusion protein region and nucleoprotein region of 15 BPI3 UK isolates identified two distinct genotypes as has been previously described elsewhere [28]. Our observations suggest that the substitutions are largely non-synonymous, suggesting the sequence variation is likely to be of limited antigenic or clinical significance (data not shown; representative of BPI3i and BPI3ii sequences deposited with Genbank as JQ627625 and JQ627626 respectively). As the gene regions targeted in the current assay were not assigned to the BPI3 genotypes in the sequence databases at the time of assay design they are referred to herein as BPI3 i & ii. Two clinical isolates L3380 (BPI3 i) and A2112 (BPI3 ii) were used for optimisation of the assay. A further three isolates each of BPI3 i and ii strains were used for preliminary validation. The various viruses used for technical validation of the mRT-qPCR are shown in Table 1.

### Respiratory viral culture

Samples were processed in primary bovine embryonic kidney cells (BEK) using standard methods. Briefly, the tissues were homogenised using a gentleMACS dissociator with M tubes (both Miltenyi Biotec Ltd. Surrey, UK) in virus transport medium (VTM; Hanks balanced salt solution containing 0.02% phenol red, 1% w/v bovine albumin, 0.45 w/v sodium hydrogen carbonate, 600 u/ml benzyl penicillin, 0.3 mg/ml streptomycin sulphate, 50 u/ml polymyxin B and 50 u/ml nystatin), centrifuged at 2,000 × g for 10 min at 4°C, filtered through 0.45 nm filter and the supernatant was harvested for inoculating primary bovine embryo kidney (BEK) cell culture. For respiratory swabs and bronchoalveolar lavage (BAL) samples in VTM, the entire sample was sonicated for 30 s, filtered through 0.45 nm filter and the supernatant was harvested for inoculation. 200 μl of each sample was added to cell cultures maintained in tubes, incubated at 37°C, 5% CO_2 _and observed for the development of cytopathic effect (CPE) for 5-7 days. Two passages were routinely performed.

### mRT-qPCR specificity

The specificity of the mRT-qPCR was assessed by testing viral nucleic acid extracted from a range of veterinary viruses, which had been maintained in cell culture and validated in other diagnostic tests, comprising border disease virus, bovine viral diarrhoea virus, bovine adenovirus, ovine adenovirus, canine distemper virus, bovine herpesvirus 2, 4 and 6, alcelaphine herpesvirus 1, ovine parvovirus, ovine reovirus, Semliki forest virus and orf virus. Additionally, the closely related viruses human respiratory syncytial virus (a gift from the late Prof. C.A.Hart, University of Liverpool) and avian metapneumovirus (a gift from Prof R.C. Jones, University of Liverpool) were included in specificity testing.

### Clinical samples

From December 2009 to April 2011, 541 clinical samples from respiratory or abortion material (136 swabs, 19 BALs, 106 lung, 189 foetal liver, 9 spleen, 32 placenta, 19 trachea, 1 brain, 1 oesophagus, 1 tonsil, 7 foetal fluids, 6 formalin fixed paraffin embedded [FFPE] tissues and 15 cell culture supernatants) were tested by routine diagnostic tests for respiratory viruses. An aliquot from each sample was stored at -80°C for later mRT-qPCR analysis.

### RNA extraction

Total sample RNA was extracted using commercial kits as appropriate to the sample type; QIAamp Viral RNA Mini spin protocol (Qiagen, Crawley, UK) was used for samples of low cellularity (swabs and BALs), RNeasy Mini kit (Qiagen, Crawley, UK) was used for fresh or frozen tissue samples and RecoverAll™ Total Nucleic Acid Isolation Kit (Applied Biosystems, Warrington, UK) was used for FFPE sections. All methods were used as per the manufacturer's instructions. We have previously demonstrated that the QIAamp Viral RNA kit co-purifies BoHV-1 DNA from low cellularity samples [29] and, as BoHV-1 produces viral RNA during replication in tissues, for simplicity of clinical sample preparation we did not explicitly extract DNA for BoHV-1 detection. Phosphate buffered saline (PBS) was used as an extraction control for all extraction methods. All nucleic acid extractions were stored at -80°C prior to testing.

### Preparation of RNA controls

Plasmids containing the mRT-qPCR target sequences for BRSV, BPI3 and BoHV-1 were produced using the TOPO TA cloning kit (Invitrogen) and RNA transcribed *in vitro *(Riboprobe system, Promega) according to manufacturers' protocols. RNA quality and integrity was confirmed using a RNA NanoLabChip on an Agilent 2100 Bio analyser (Agilent Technologies) and used to construct standard curves from 10^0 ^to 10^10 ^copies. All samples had an RNA integrity number greater than 9.

### Primers, probes and mRT-qPCR

The primer and probe sequences, fluorophores and quenchers used are shown in Table 6[22,30]. As appropriate fluorophore combinations could not be designed using MGB probes to allow multiplexing with four colour detection, the reporter dyes were altered and the BRSV probe was modified to replace the minor groove binding (MGB) modification by locked nucleic acids (LNA). A β-actin primer and probe set was also used in a separate mono-specific assay to assess sample integrity and mRT-qPCR inhibition.

**Table 6:** Primers and probes for multiplex real-time RT-PCRa

**Viruses**	**Primers/probes**	**Sequences (5'-3')**
BPI3 i	BPI3euro Forward	GGTAGGAGCACCTCCACGATT
	
	BPI3euro Reverse	GCTCCAAGGCATGCTGGATA
	
	BPI3euroMGB	VIC-AAGATCTTGTTCACACATTC-MGB-NFQ

BPI3 ii	BPI3 Forward2	TGATTGGATGTTCGGGAGTGA
	
	BPI3Reverse2	AGAATCCTTTCCTCAATCCTGATATACT
	
	BPI3Fam	FAM-TACAATCGAGGATCTTGTTCA-MGB-NFQ

BRSV [14]	BRSVn Forward	GGTCAAACTAAATGACACTTTCAACAAG
	
	BRSVn Reverse	AGCATACCACACAACTTATTGAGATG
	
	BRSVLNACyan500	Cyan500-TAGTACAGGTGACAA+CA+T+TG-BBQ

BoHV-1 [15]	gB Forward	TGTGGACCTAAACCTCACGGT
	
	gB Reverse	GTAGTCGAGCAGACCCGTGTC
	
	gBCy5	Cy5-AGGACCGCGAGTTCTTGCCGC-BHQ2

β-actin	Bac1F_uni	GACAGGATGCAGAARGAGATCAC
	
	Bac2R_uni	TCCACATCTGCTGGAAGGTG
	
	β-actin FAM-MGB	FAM-TGAAGATCAAGATCATCG-MGB-NFQ

^a ^All the primers and probes are obtained from Applied Biosystems (Warrington, UK) except BRSV LNAcyan500 probe (TIB Molbiol,Berlin, Germany) and gBCy5 probe (Eurofins MWG Operon, Germany).

Abbreviations: *BHQ *black hole quencher, *MGB-NFQ *minor groove binding-non fluorescent quencher, *LNA *locked nucleic acid, *BBQ *Blackberry quencher.

### mRT-qPCR conditions

Optimisation of the mRT-qPCR included prior assessment of the mono-specific assays, testing several master mix reagent sets, titration of primer and probe concentrations and alteration of RT and PCR cycling parameters. Quantifast Multiplex RT-PCR (Qiagen, Crawley, UK) was chosen as the optimum reagent for mRT-qPCR. Reactions were performed in triplicate on 96 well plates (Lightcycler 480 multiwell plate 96, Roche UK) in a total volume of 25 μl containing a final concentration of 200 nM each primer and 100 nM each probe and 1 μl of template RNA. The β-actin mono-specific RT-PCR was performed in a similar manner except that final primer and probe concentrations were 500 nM and 200 nM respectively.

All reactions performed on a Light Cycler 480 II (Roche UK) with the following cycling parameters; 50°C for 20 min (RT) and 95°C for 2 min (hot start), 40 cycles of 95°C for 15 s (denaturation) and 60°C for 1 min (annealing/extension), followed by a final stage of 40°C for 10 s (cooling). The results were analysed using Light Cycler 480 SW1.5 software (Roche UK). For each amplification plot, a crossing point value (Cp), representing the PCR cycle number at which the reporter dye fluorescence was detectable above the background fluorescence, was calculated automatically using the '2nd derivative max' method. Cross-talk was eliminated almost entirely by applying colour compensation as per the protocol provided by the instrument manufacturer (Roche UK); the use of the four fluorophores allowed differentiation of the virus targets.

### Simultaneous detection of multiple virus targets

A template mix including ten-fold serial dilution of the four *in vitro *transcribed virus targets was tested.

### Statistical analysis

A linear mixed model was fitted to the observed Cp values of each virus. The day of experiment was fitted as a random effect while the logarithm (base 10) of viral copy number and its polynomial terms (quadratic and cubic), RT-PCR method (two levels: uniplex and multiplex), and the interaction effect of RT-PCR method and log viral copy number were fitted as fixed effects. The heterogeneity of variance due to different levels of viral copy number was also considered. The estimates of variance were obtained using the restricted maximum likelihood (REML) method. Only fixed main effects and interaction terms that were statistically significant (*P *≤ 0.05) were retained in the final model. Standard model assumptions of normality and independence of error terms were also assessed. The estimates of means and variance components obtained from the final fitted model were then used to quantify the statistical properties of Cp estimates using a parametric bootstrap sampling process [31]. For each virus and choice of log copy number, the model fitted to the original data was used to generate a total of 1000 random pseudo-test samples within each bootstrap cycle, and the bootstrap process was repeated 10,000 times. The outcomes of all simulated data were used to estimate the mean cut-off value of Cp that equates to 97% sensitivity for the diagnostic test for each specific virus, conditional on the log viral copy number and the RT-PCR method, while 95% confidence intervals for these estimates were calculated using 10,000-term bootstrap distribution of each quantity.

Estimates of repeatability standard deviation (SD_r_) and relative standard deviation (RSD_r_) for each assay were calculated as suggested by Horwitz [32]. To estimate the repeatability of an assay for each virus, linear models were fitted to the uniplex and multiplex assay data separately. The estimates of SD_r _and corresponding 95% confidence intervals for each assay were obtained from the estimate of residual standard deviation of the model. The estimate of RSD_r _(or coefficient of variation) of repeatability was obtained as the ratio of SD_r _to mean and expressed as a percentage. All statistical analyses were carried out using R software version 2.13.1 [33].

## Authors' contributions

LT participated in the initiation, conception and planning of work, execution of work and writing of the manuscript, MM carried out some of the assays for reproducibility, JG was instrumental in organising the sample preparation for the assay, TC optimised the BoHV-1 PCR as a mono-specific assay and supplied samples from aborted fetuses, EL and DT carried out the RNA extractions, MN performed the statistical analysis and KW conceived the study and participated in the design and coordination and critically reviewed the manuscript. All authors read and approved the final manuscript.
